# Chemical Profiles and Simultaneous Quantification of *Aurantii fructus* by Use of HPLC-Q-TOF-MS Combined with GC-MS and HPLC Methods

**DOI:** 10.3390/molecules23092189

**Published:** 2018-08-30

**Authors:** Yingjie He, Zongkai Li, Wei Wang, Suren R. Sooranna, Yiting Shi, Yun Chen, Changqiao Wu, Jianguo Zeng, Qi Tang, Hongqi Xie

**Affiliations:** 1 Hunan Key Laboratory of Traditional Chinese Veterinary Medicine, Hunan Agricultural University, Changsha 410128, China; yingjiehe272@163.com (Y.H.); zengjianguo@hunau.edu.cn (J.Z.); 2 National and Local Union Engineering Research Center for the Veterinary Herbal Medicine Resources and Initiative, Hunan Agricultural University, Changsha 410128, China; 18390946378@163.com (W.W.); erin643747964@163.com (Y.S.); m18250068565_1@163.com (Y.C.); m15207494911@163.com (C.W.); 3 School of Medicine, Guangxi University of Science and Technology, Liuzhou 565006, China; m13657294483_1@163.com; 4 Department of Surgery and Cancer, Chelsea and Westminster Hospital, Imperial College London, London SW10 9NH, UK; s.sooranna@imperial.ac.uk

**Keywords:** *Aurantii fructus*, HPLC-Q-TOF-MS, GC-MS, HPLC, biochemical markers, quality control

## Abstract

*Aurantii fructus* (AF) is a traditional Chinese medicine that has been used to improve gastrointestinal motility disorders for over a thousand years, but there is no exhaustive identification of the basic chemical components and comprehensive quality control of this herb. In this study, high-performance liquid chromatography coupled with quadrupole time of flight mass spectrometry (HPLC-Q-TOF-MS) and gas chromatography coupled mass spectrometry (GC-MS) were employed to identify the basic chemical compounds, and high-performance liquid chromatography (HPLC) was developed to determine the major biochemical markers from AF extract. There were 104 compounds belonging to eight structure types, including 13 amino acids or peptides, seven alkaloids, 18 flavanones, 14 flavones, 15 polymethoxyflavonoids, six triterpenoids, nine coumarins, and 18 volatile oils, as well as four other compounds that were systematically identified as the basic components from AF, and among them, 41 compounds were reported for the first time. Twelve bioactive ingredients were chosen as the benchmark markers to evaluate the quality of AF. The analysis was completed with a gradient elution at a flow rate of 0.7 mL/min within 55 min. This efficient method was validated showing good linearity, precision, stability, repeatability and recovery. Furthermore, the method was successfully applied to the simultaneous determination of 12 chemical markers in different samples of AF. This study could be applied to the identification of multiple bioactive substances and improve the quality control of AF.

## 1. Introduction

*Aurantii fructus* (AF) is the dried and immature fruit of *Citrus aurantium* L. and its cultivars. It plays important roles in traditional Chinese medicine (TCM) and is a functional food that has been intensively applied to treat stagnation of dyspepsia, improve gastrointestinal motility dysfunction, and alleviate chest pain in traditional therapies [1,2]. There is a general consensus that the pharmacological effects of herbal medicines are significantly correlated with the chemical composition and the contents of bioactive compounds in herbs. The current research shows that the main bioactive constituents in AF are mainly flavonoids [2,3,4], alkaloids [1,4,5], coumarins [6,7], and volatile oils [1,8]. Modern pharmacological studies have shown these compounds to exhibit various activities, e.g., anti-inflammatory [9,10], anti-oxidation [10,11], regulation of gastrointestinal and prevention of cardiovascular disease [12,13], and they are considered to be the major bioactive constituents in AF. Plenty of clinical investigations have shown that the main pharmacological ingredients of AF are flavonoids, such as flavanones and polymethoxyflavonoids (PMFs), that mainly focus on regulating gastrointestinal dysmotility [12,14,15].

The chemical compounds from AF have been qualitatively or quantitatively analyzed via ultraviolet spectrophotometry (UV), thin layer chromatography (TLC), gas chromatography (GC), high performance liquid chromatography (HPLC), gas chromatograph-mass spectrometry (GC-MS), liquid chromatography mass spectrometry (LC-MS), infra-red spectrum (IR), and nuclear magnetic resonance (NMR) [1,2,7,16,17]. However, due to the complexity of the structural types of the basic chemical constituents of TCM, these methods have certain deficiencies in the analysis of the basic components, and there are incomplete reports for a comprehensive investigation on the constituents of AF. In particular, some ingredients are found in low sensitivity and trace amounts of the above methods often lead to insufficient and defective analysis. High-performance liquid chromatography quadrupole time-of-flight mass spectrometry (HPLC-Q-TOF-MS) is an efficient method with high sensitivity, high precision, high resolution, fast information acquisition, and has been employed to analyze complicated profiles of citrus plants in recent years [6,18,19]. Therefore, the detailed chemical constituents of AF extract can be identified systematically using the HPLC-Q-TOF-MS method. Alternatively, volatile components with low content can be identified by GC-MS technique. 

TCM plays a clinical therapeutic role by applying multiple constituent works on multiple targets within the body [20], but only two flavanones (naringin, neohesperidin) are defined as the evaluated markers of quality control for AF in the Chinese Pharmacopoeia [21]. Due to the comprehensive pharmacological effects of TCM, the evaluated standards of quality control for TCM should be carefully considered and established based on multiple pharmacodynamic substances in the future. 

The aim of this study is to establish a new comprehensive analysis method for gaining insight into the exhaustive chemical profiles of AF using HPLC-Q-TOF-MS combined with GC-MS. Then, in consideration of the major pharmacological effects of AF, the typical bioactive ingredients were successfully screened as the markers of quality control from the complicated compounds of AF based on the analytical data from mass spectrometry. Then, a simple and effective method for simultaneous determination of bioactive markers was established by high performance liquid chromatography (HPLC), and AF samples from different regions were evaluated. To the best of our knowledge, this is the first attempt to improve qualitative and quantitative methods for the analysis and determination of bioactive ingredients in AF with efficient procedures [22]. These results supply complete chemical components for further research and improve the practical application of the consistency evaluation and quality control of AF samples from different habitats. In addition, they provide comprehensive references for the research and development of AF to be used as a traditional medicine and functional food.

## 2. Results and Discussion

### 2.1. Identification and Analysis of Active Constituents in Ethanol Extract of AF

**Figure 1:**
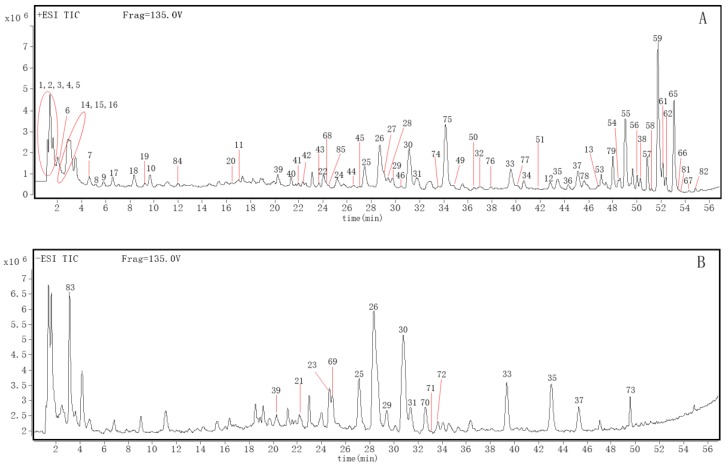
Total ion chromatography of ethanol extract from *Aurantii fructus* in positive (**A**) and negative (**B**) ion modes.

As many as 86 compounds were identified as the main constituents from AF by ESI-Q-TOF-MS in the positive and negative ion mode, and the total ion chromatogram was analyzed based on the standards, fragmentation patterns, literature and the ChemSpider database (Figure 1). The retention time and mass spectrometry information of each chemical constituent of AF were detected as shown in Table 1. Eighty-six compounds in the ethanol extract of AF were accurately identified or preliminarily assigned. These compounds included 13 amino acids or peptides, seven alkaloids, 18 flavanones, 14 flavones, 15 polymethoxyflavonoids, six triterpenoids, nine coumarins, and four other compounds. The structures of these compounds were drawn and classified by ChemBio Draw Ultra 14.0, as shown in Figure S1. Thirty-seven of these compounds were reported in AF for the first time.

#### 2.1.1. Amino Acids and Polypeptides

Amino acids are important nutrients needed by the human body and are major primary metabolites of citrus plants [23], as well as the basic substrates in the biosynthesis of secondary metabolites of many plants. Although various free amino acids can be detected in different species of citrus, such as lemon, orange and other fruits [23,24], at present there is still no reports on the amino acid composition of AF. Owing to the low content and larger polarity of amino acids, it was difficult to obtain their MS^2^ spectra. However, based on the high resolution and accuracy of Q-TOF-MS and the accuracy of the measured molecular weight in the MS data, multiple types of amino acids were found in AF. 

Compound **1** displayed a protonated molecule at *m*/*z* 147.1130 [M + H]^+^ with the molecular formula C_6_H_14_N_2_O_2_. From the MS data (Table 1), compound **1** corresponds to lysine, which was isolated previously from citrus fruits [23]. Compound **2** showed a protonated molecule at *m*/*z* 147.0748 [M + H]^+^ with the molecular formula C_5_H_10_N_2_O_3_. Compound **2** was presumed tentatively as glutamine [23]. By use of the same method and mass spectrometer formula prediction software (absolute error ˂5 ppm), combined with literature references, compounds **3**, **4**, **5**, **6**, **7**, **8**, **9**, **10** and **11** can be identified tentatively as histidine [23], arginine [23,24], proline [23,24], valine [23], isoleucine [23], leucine [23], tyrosine [23], phenylalanine, and tryptophan [23], respectively (Table 1). In addition, two polypeptides were found in AF. Compound **12** displayed a protonated molecule at *m*/*z* 728.3966 [M + H]^+^ with the molecular formula C_36_H_53_N_7_O_9_, and corresponded to citrusin III [25,26,27], isolated previously from citrus plants. Compound **13** showed a protonated molecule at *m*/*z* 704.3978 [M + H]^+^ with the molecular formula C_34_H_53_N_7_O_9_, which is consistent with the literature, and presumed as citrusin I [26,27]. These 13 compounds were reported in AF for the first time.

#### 2.1.2. Alkaloids

Compound **17** displayed a protonated molecule at *m*/*z* 168.1016 [M + H]^+^ with the molecular formula C_9_H_13_NO_2_, and MS/MS fragmentation showed peaks at *m*/*z* 150.0853 [M + H]^+^. The fragmentation pattern of compound **17** agreed with synephrine [28,29], and it was unambiguously identified by comparison with the standard. Compound **18** showed a protonated molecule at *m*/*z* 152.1071 [M + H]^+^ with the molecular formula C_9_H_13_NO, which is consistent with the literature, and presumed to be *N*-methyltyramine [28,29]. Compound **19** displayed a protonated molecule at *m*/*z* 268.1027 [M + H]^+^ with the molecular formula C_10_H_13_N_5_O_4_, which is consistent with the literature, and presumed to be adenosine [30]. Compound **20** showed a protonated molecule at *m*/*z* 196.0970 [M + H]^+^ with the molecular formula C_10_H_13_NO_3_, which is consistent with the literature, and presumed to be *N*-acetylnorsynephrine [29]. In addition, other alkaloids (absolute error ˂5 ppm) were analyzed in this experiment. The protonated molecules of compounds **14**, **15** and **16** were *m*/*z* 138.0549 [M + H]^+^, *m*/*z* 130.0865 [M + H]^+^, and *m*/*z* 144.1022 [M + H]^+^, respectively. Due to the high solution of Q-TOF-MS, these compounds were preliminarily assigned to aminobenzoic acid, pipecolic acid, and piperidine acetic acid, respectively. These three compounds were reported in AF for the first time. 

#### 2.1.3. Flavonones

Flavonones are considered to be the most important compounds obtained from AF. As many as 18 flavonones were identified by ESI-Q-TOF-MS in this study (Table 1), and among these flavonones, compounds **22**, **24**, **25**, **26**, **29**, **30**, **33**, **35**, **37** were unambiguously identified as eriodictyol-7-*O*-rutinoside (eriocitrin) [1,2], eriodictyol-7-*O*-neohesperidoside (neoeriocitrin) [2,31], naringenin-7-*O*-rutinoside (narirutin) [1,2,31], naringenin-7-*O*-neohesperidoside (naringin) [2,30,31], hesperetin-7-*O*-rutinoside (hesperidin) [2,30,31], hesperetin-7-*O*-neohesperidoside (neohesperidin) [1,2,31], isosakuranetin-7-*O*-neohesperidoside (poncirin) [1,2], naringenin [2], and hesperetin [2], respectively, by comparison of the retention time, absorption wavelengths, and *m*/*z* values with the reference standards and the literature. 

The remaining compounds could be tentatively assigned by comparing the accurate mass data (absolute value of error <5 ppm), the formula predictor software, the fragmentation patterns, and the literature data (Table 1). For example, in the negative mode, compounds **21** and **23** had *m*/*z* 741.2261 [M − H]^–^ and *m*/*z* 449.1101 [M − H]^–^, respectively, and the chemical formula were C_33_H_42_O_19_ and C_21_H_22_O_11_, respectively. They were presumed to be naringenin-7-*O*-triglycoside [2,31] and eriodictol-7-*O*-glucoside [32] according to literature. In the positive mode, Compounds **27**, **31** and **34** had *m*/*z* 435.1286 [M + H]^+^ with the molecular formula C_21_H_20_O_10_, *m*/*z* 465.1381 [M + H]^+^ with the molecular formula C_22_H_24_O_11_, and *m*/*z* 449.1432 [M + H]^+^ with the molecular formula C_22_H_24_O_10_, respectively. Their parent nucleus were *m*/*z* 273.0751 [M + H]^+^, *m*/*z* 303.0861 [M + H]^+^, *m*/*z* 287.0898 [M + H]^+^, respectively, suggesting that they contained glucoside (162 Da). According to the fragmentation patterns of naringin (**26**), neohesperidin (**30**) and poncirin (**33**), compounds **27**, **31** and **34** were tentatively identified as naringenin-7-*O*-glucoside [33], hesperetin-7-*O*-glucoside [33], and isosakuranetin-7-*O*-glucoside (isosakuranin) [34], respectively (Table 1). Similarly, compounds **32** and **38** were presumed to be eriodictyol [35] and isosakuranetin [35]. Compound **28** displayed protonated and deprotonated molecules at *m*/*z* 755.2392 [M + H]^+^ and *m*/*z* 753.2233 [M − H]^–^, respectively, with the molecular formula C_34_H_42_O_19._ It was assigned tentatively to brutieridin [28,36]. Compound **36** showed protonated and deprotonated molecules at *m*/*z* 725.2285 [M + H]^+^ and *m*/*z* 723.2127 [M − H]^–^, respectively, with molecular formula C_33_H_40_O_18_. It was assigned tentatively to melitidin [28,36]. Among them, naringenin, hesperetin, eriodictyol and isosakuranetin were identified as the basic nucleus, and flavonones in AF were deduced to be generated based on these nuclei. Compounds **23**, **28**, **34** and **36** were reported in AF for the first time.

#### 2.1.4. Flavones

A total of 14 flavones were identified or preliminarily assigned in the study. Compound **39** was accurately identified as quercetin-3-*O*-rutinoside (rutin) [2] by a reference standard and mass spectrometry. Compounds **40** and **51** displayed protonated molecules at *m*/*z* 595.1663 [M + H]^+^ with molecular formula C_27_H_30_O_15_ and at *m*/*z* 697.1964 [M + H]^+^ with molecular formula C_31_H_36_O_18_, were assigned to isovitexin-7-*O*-glucoside (saponarin) [31,32], and isovitexin-7-*O*-xylocoside-2″-*O*-arabi-noside [31], respectively. Compounds **43**, **46** and **50** contained protonated molecules at *m*/*z* 741.2213 [M + H]^+^, *m*/*z* 579.1704 [M + H]^+^ and *m*/*z* 579.1706 [M + H]^+^, respectively. With the same MS^2^ fragment ion at *m*/*z* 271 [M + H]^+^ for these three compounds, it was identified to be apigenin (**52**) [35] as a nucleus structure according to the literature data. In positive ion mode, it was presumed that the two compounds contained rutinoside or neohesperidoside because of the low weight of 308 Da, and the abundance of the protonated molecule at *m*/*z* 271 [M + H]^+^ in compound 46 was lower than compound **50**. Based on the above information and literature reports [2,27], it was speculated that compounds **46** and **50** were apigenin-7-*O*-rutinoside (isorhoifolin) [28] and apigenin-7-*O*-neohesperidoside (rhoifolin) [28], and compound **43** was preliminarily assigned to apigenin-7-*O*-rutinoside-4′-*O*-glucoside (isorhoifolin-4′-*O*-glucoside) [37]. Compounds **41** and **42** contained the same protonated molecules at *m*/*z* 625 [M + H]^+^ with the molecular formula C_28_H_32_O_16_, and were presumed to be isomers of diosmetin-6,8-*di-C*-glucoside [28] according to the literature. Compounds **44**, **47**, **48** contained a same MS^2^ ion fragment of *m*/*z* 301 [M + H]^+^, and their protonated molecules were at *m*/*z* 463.1240 [M + H]^+^, *m*/*z* 609.1802 [M + H]^+^, and *m*/*z* 609.1807 [M + H]^+^, respectively. According to the previous fragmentation patterns combined with literature reports, these four compounds were speculated to be diosmetin-7-*O*-glucoside, diosmetin-7-*O*-rutinoside (diosmin) [28], and diosmetin-7-*O*-neohesperidoside (neodiosmin) [28], respectively. Compound **45** was tentatively assigned to luteolin-7-*O*-rutinoside (veronicastroside) [36,37]. Compound **49** displayed protonated molecules at *m*/*z* 667.1886 [M + H]^+^ with molecular formula C_30_H_34_O_17_, and it was speculated to be diosmetin-7-*O*-(6″-*O*-acetyl) neohesperidoside according to the MS data (Table 1). The compounds **42**, **43**, **44**, **45**, **49, 51** were reported in AF for the first time.

#### 2.1.5. Polymethoxyflavonoids

Polymethoxyflavonoids (PMFs) are based on the core aglycone structure that is modified by different numbers and/or positions of methoxyl/hydroxyl substituents [18]. The molecular weights of PMFs can be calculated in advance by adding *n* × 30 and/or *n* × 16 according to the basic flavone structure at 222 Da. Then, the chemical formula and the corresponding molecular weight of every possible PMF isomer can be designated, which is very helpful in speculating the structures of PMFs in the complex extracts of AF.

In the study, PMFs were analyzed in the positive ion mode due to the ideal protonated effect for these compounds. Compounds **59** and **65** were identified undisputed as 5,6,7,8,3′,4′-hexamethoxyflavone (nobiletin) [2,4] and 5,6,7,8,4′-pentamethoxyflavone (tangeretin) [2,4], respectively, based on the standard substances, retention times, accurate molecular weight and fragmentation patterns. Generally, these compounds tend to lose *n*CH_3_**·** and produce the ion fragment [M + H − *n* × 15]^+^, and on this basis, the molecular weights that are easy to lose include CH_4_ (16 Da), H_2_O (18 Da), CO (28 Da), CH_4_ + CH_3_ (31 Da), H_2_O + CH_3_·(33 Da), CO + CH_3_ (43 Da), CO_2_ (44 Da), H_2_O + CO (44 Da) and CO + H_2_O + CH_3_ (59 Da) [18,38]. For example, tangeretin contained the protonated molecules at *m*/*z* 373 [M + H]^+^ with molecular formula C_28_H_32_O_16_. In addition, prominent ions at *m*/*z* 358.1000 [M + H-CH_3_]^+^, *m*/*z* 343.0722 [M + H-2CH_3_]^+^, *m*/*z* 325.0655 [M + H-2CH_3_-H_2_O]^+^, and *m*/*z* 297.0697 [M + H-2CH_3_-H_2_O-CO]^+^ show a loss of 15 Da, 30 Da, 48 Da, and 76 Da, respectively. 

According to the fragmentation patterns of PMFs, the literature, and the MS data (Table 1), 3-hydroxy-5,7,8-trimethoxyflavone (**53**) [18], 5-hydroxy-6,7,3′,4′-tetramethoxyflavone (**54**) [2,39], 5-hydroxy-6,7,3′,4′,5′-pentamethoxyflavone (**55**) [2,18], 5,7,8,3′,4′-pentamethoxyflavone (**56**) (isosinensetin) [2,18], 5,6,7,3′,4′-pentamethoxyflavone(**57**) (sinensetin), 5-hydroxy-6,7,8,3′,4′-pentamethoxyflavone(**58**) [18,39], 3,5,6,7,8,3′,4′-heptamethoxyflavone (**60**) [2,18], 5,6,7,4′-tetramethoxyflavone (**61**), 5-hydroxy-3,6,7,8,3′,4′-hexamethoxyflavone (**62**) [18], 5-hydroxy-3,6,7,8-tetramethoxyflavone (**63**) [18], 5,7,8,4′-tetramethoxyflavone (**64**) [18,39], 3-hydroxy-5,6,7,8,3′,4′-hexamethoxyflavone (**66**) (natsudaidai) [7,39], and 5-hydroxy-6,7,8,4′-tetramethoxyflavone (**67**) [7,39], were identified or assigned tentatively. Compounds **53**, **55**, **62** and **63** were reported in AF for the first time.

#### 2.1.6. Triterpenoids

Triterpenoids, especially limonoids, are often reported in citrus plants. Studies have shown that they have good anti-tumor activity, and have antifeedant activity to insects [40]. A total of six triterpenoids were identified or speculated in AF by Q-TOF-MS analysis. Compound **68** was identified certainly as limonin [41] by comparing with a reference substance, the retention time and the precise molecular weight. Compound **73** was speculated to be an isomer of limonin [41] due to the same protonated molecule at *m*/*z* 471 [M + H]^+^ with the molecular formula C_26_H_30_O_8_. Compound **69** showed a deprotonated molecule at *m*/*z* 649.2482 [M − H]^−^ with chemical formula C_32_H_42_O_14_, which was consistent with the literature, and presumed to be limonin-17-*β-*d-glucoside [41,42]. Similarly, Compound **70** displayed a deprotonated molecule at *m*/*z* 693.2753 [M − H]^−^ with the chemical formula C_34_H_46_O_15_, and it can be presumed to be nominin-17-*β-*d-glucoside [42]. Compounds **71** and **72** were assigned tentatively to obacunoic acid-17-*β-*d-glucoside [42] and nomilinic acid-17-*β-*d*-*glucoside [41,42], respectively. Compounds **70**, **71** and **73** were reported in AF for the first time.

#### 2.1.7. Coumarins

A sum of nine compounds was identified or tentatively speculated as coumarins in the study. Compounds **76** and **82** were identified accurately as xanthotoxol [32,43] and auraptene [32], respectively, based on retention times, accurate molecular weights and comparison with reference substances. Compound **74** was preliminarily deduced as phellopterin [32,43] at a deprotonated molecule of *m*/*z* 301.1065 [M + H]^+^ with the chemical formula C_17_H_16_O_5_. Compound **75** and **80** contained the same deprotonated molecule (*m*/*z* 261 [M + H]^+^) with the chemical formula (C_17_H_16_O_5_), and they were identified tentatively as meranzin [44] and isomeranzin [36], respectively. Similarly, compounds **77**, **78**, **79** and **81** were tentatively identified as oxypeucedanin [6,32,43], scopoletin [34], epoxybergamottin [6,43], and osthol [6,32,43], respectively, based on MS information and literature data. Compounds **75**, **78** were reported in AF for the first time.

#### 2.1.8. Other Compounds

Compound **83** showed a deprotonated molecule at *m*/*z* 191.0188 [M − H]^−^ with chemical formula C_6_H_8_O_7_, and was identified as citric acid [4,30], which commonly exists in citrus plants. Compound **84** displayed a protonated molecule at *m*/*z* 127.0388 [M + H]^+^ and MS^2^ ions at *m*/*z* 109.0289 [M + H]^+^, and was tentatively deduced to be 5-hydroxymethyl furfual [37]. Compound **85** showed a protonated molecule at *m*/*z* 481.1680 [M + H]^+^ with the chemical formula C_23_H_28_O_11_, and was assigned to paeoniflorin [31]. Compound **86** displayed a protonated molecule at *m*/*z* 625.2100 [M + H]^+^ with the chemical formula C_29_H_36_O_15_, and was tentatively deduced as magnoloside A [30]. Compound **84** and **85** were reported in AF for the first time.

### 2.2. Analysis of the Constituents of Volatile Oils Obtained from AF

**Figure 2:**
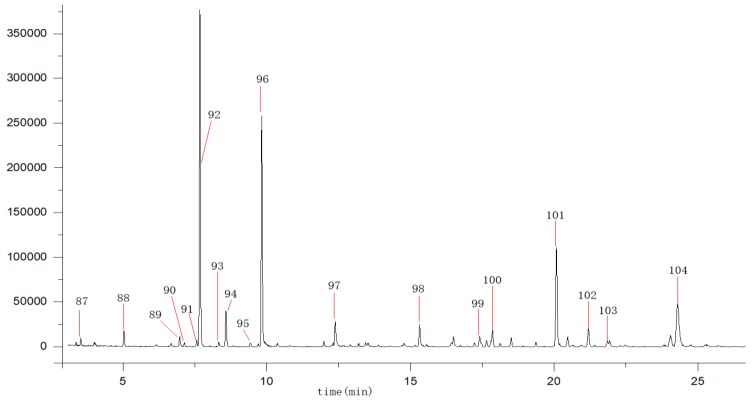
Total ion chromatgraphy of the volatile oils from *Aurantii fructus.*

The volatile components of AF were extracted by supercritical fluid extraction technology (SFE), which is widely used in the extraction of low-pole components with the advantages of low energy consumption, no pollution, high extraction rate and good product purity. Separation of particular volatile components was carried out by means of the optimal GC-MS method, as shown in Figure 2. Then, the AF volatiles were identified by the comparison of their mass spectra with those recorded in the National Institute of Standards and Technology mass spectral library (NIST05a.L). A total of 18 volatile compounds [1,8] were identified, as shown in Table 2. The structures of these compounds were drawn as shown in Figure S2.

The data in Table 2 illustrate the relative amount of each component detected in the AF volatiles (calculated with their relative peak areas). *d*-limonene (**92**) and linalool (**96**) were identified as the main aromatic components. In additional, volatile constituents such as (−)-*α*-pinene (**88**), α-Phellandrene (**89**), 3-carene (**90**), Ocimene (**93**), 4-carene (**94**) Cyclohexene (**95**), and terpineol (**97**) were commonly found in AF by comparing literature data [1,8]. Four volatile constituents included thymol (**98**), copaene (**100**), 1.6-cyclodecadiene (**101**) and (+)-aromadendrene (**103**), which were identified in AF for the first time. 

### 2.3. Selection of the Quantitative Chemical Markers from AF

The effective constituents obtained from AF are complex and various, according to a previous qualitative study. For the selection of quantitative chemical markers, four main principles are followed [45]. First, they are components absorbed in vivo. Generally, the components absorbed in vivo are considered as potential directly effective materials with therapeutic effects. Second, the quantitative markers should also exhibit the same or similar activity as indicative of the TCM. Third, in consideration of the quality control of TCM, quantitative markers should be found from different individual samples in the TCM. Another important point is that the quantitative markers of different samples should be taken into account to ensure the improvement of practicality, based on the Chinese Pharmacopoeia (2015 edition).

In previous studies, flavanones such as naringenin, naringin, narirutin, neohesperidin, hesperidin, neoeriocitrin and poncirin were identified as the main bioactive components absorbed in rat plasma after oral administration of AF extract [5,46,47]. Moreover, PMFs, such as nobiletin and tangeretin, were also absorbed as constituents, because they were found in rat plasma [16,46]. Many investigations have explored that the main pharmacological effects of these flavonoids, and mainly focus on regulating gastrointestinal dysmotility [12,14,15], which was consistent with the traditional clinic application of AF. These compounds were easily detected from different samples of AF, but only naringin and neohesperidin are defined as the chemical markers in the Chinese Pharmacopoeia (2015 edition). Hence, the quality control for AF should be comprehensively considered and established based on multiple pharmacodynamic substances. Except for naringin and neohesperidin, ten other components (eriocitrin, neoeriocitrin, narirutin, hesperidin, poncirin, naringenin, hesperetin, nobiletin, tangeretin, auraptene) were selected as quantitative chemical markers in order to establish a comprehensive quality control for AF.

#### 2.3.1. Optimization of Chromatographic Conditions

Due to the variety and complexity of the constituents in AF, it is necessary to establish a chromatographic separation method with specificity for the analysis of all the chemical markers. Therefore, several experimental parameters that influence the separation performance of the proposed HPLC method were investigated and optimized in the study.

**Figure 3:**
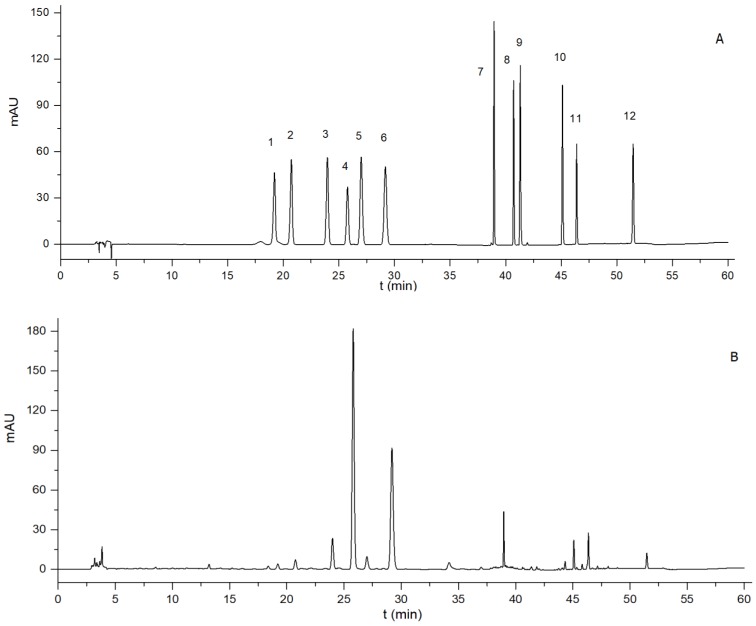
HPLC chromatograms of 12 chemical markers (**A**) and an *Aurantii fructus* sample (**B**). (1. eriocitrin, 2. neoeriocitrin, 3. narirutin, 4. naringin, 5. hesperidin, 6. neohesperidin, 7. poncirin, 8. naringenin, 9. hesperetin, 10. nobiletin, 11. tangeretin, 12. auraptene).

For example, acetonitrile-water was selected as the mobile phase, due to its better separation of peaks compared with methanol-water in this study, and formic acid was added in the mobile phase to improve the tail behavior of the flavonoids. Full-wavelength scanning was performed during the analysis of AF because of the difference in maximum absorption wavelength of these compounds. The main absorption wavelengths of these markers are 273 nm, 284 nm, and 325 nm, respectively. According to the analysis of preliminary experiments, the final wavelength was set to 284 nm, which exhibited good detection of these chemical markers. Moreover, the flow rate of the mobile phase, column temperature and injection volume were also optimized via pre-experience at an efficient level, and the solvents saved. These markers were well separated and directly determined, as shown in Figure 3, which was conducive to the HPLC quantitative analysis of AF.

#### 2.3.2. Methodological Verification

Calibration and verification of analytical methods are very important for quality assurance (QA) and quality control (QC) in practical applications. The feasibility of the chromatographic method for determining these bioactive markers should be evaluated via methodological verification including linearity, the limits of detection (LOD) and quantification (LOQ), precision, stability, repeatability and recovery. The analysis was performed by HPLC, and the linearity, precision, accuracy, recovery of each bioactive marker, and the stability of this method were analyzed as listed in Table 3. 

The high-correlation coefficient values (r > 0.9995) obtained indicated that there were good linear correlations between the concentrations of the relative standard deviations (RSDs) of investigated compounds and their peak areas within the test concentrations, and the obtained LODs and LOQs were less than 0.21 μg/mL and 0.67 μg/mL, respectively. The precision, repeatability, and stability for each compound was less than 1.97%, 3.50%, and 3.38%, respectively. In addition, the developed method showed good recoveries at a range of 98.35%–102.95%. The results indicated that the HPLC method was efficient, accurate, and sensitive for quantitative determination of the major chemical markers in AF.

#### 2.3.3. Quantitative Determination of the Chemical Markers in AF

**Figure 4:**
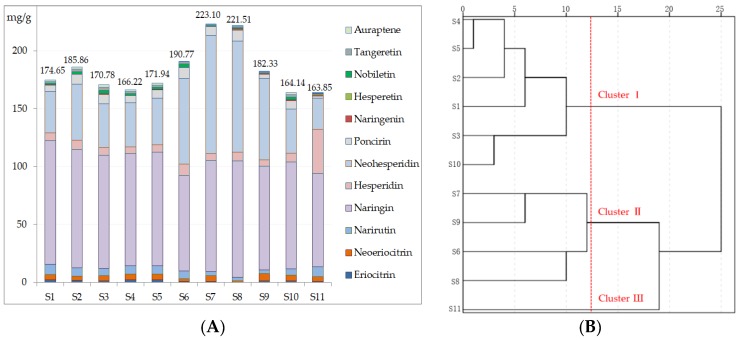
Contents of quantitative markers in *Aurantii fructus* from 11 different samples (**A**). Hierarchical clustering dendrogram of 11 samples of *Aurantii fructus* based on the quantitative analysis (**B**).

Different samples of AF (Table S1) were analyzed by the proposed method to determine the 12 chemical markers by HPLC. The quantitative results are presented in Figure 4A. Naringin and neohesperidin were the main constituents, the contents of which varied from 80.40–106.81 mg/g, and 26.97–102.3 mg/g in 11 samples, respectively. All the samples were in accordance with the standards of the Chinese Pharmacopoeia except for batch 11, because it might be a variety. In addition, it was found that narirutin, hesperidin, poncirin, and neoeriocitrin were also considerably varied from 2.87-9.15 mg/g, 5.10–38.14 mg/g, 1.80–9.40 mg/g, and 1.27–6.48 mg/g, respectively (Table S2). Meanwhile, the content of 12 markers in 11 samples had a relatively large variation. Therefore, it was recommendable to evaluate the quality in different samples of AF.

Hierarchical cluster analysis was performed based on the quantitative determination results from the different samples of AF in order to finding relatively homogeneous clusters. The contents of the 12 components in AF samples were defined as the variables in the analysis to differentiate and classify the 11 samples. In the dendrogram, the quantitative relationship reflected the degree of similarity between different AF samples. As shown in Figure 4B, 11 samples could be divided into three main clusters. Generally, samples with common contents of the compounds, such as samples 1, 2, 3, 4, 5, and 10, were arranged to be a clusters (I) in normal content range of the 12 chemical markers. Higher contents of the markers in the samples 6, 7, 8 and 9 were gathered to clusters II with high quality. Sample 11 was a single, generated as clusters III. Because of the huge difference content of the main chemical markers, naringin and neohesperidin in the sample were lower than other samples, but with a higher content of hesperidin (Figure 4A). The different samples of AF were discriminated by hierarchical cluster analysis for quality evaluation.

## 3. Materials and Methods

### 3.1. Materials, Chemicals and Reagents

Eleven samples of *Aurantii fructus* (AF) samples from different habitats were collected and identified by Associate Professor Tang Qi. Reference standards included synephrine, eriocitrin, neoeriocitrin, rutin, narirutin, naringin, hesperidin, neohesperidin, limonin, xanthotol, poncirin, naringenin, hesperetin, nobiletin, tangeretin and auraptene with a purity of over 98% were purchased from Yuan-ye Bio-Technology Co., Ltd. (Shanghai, China). Chromatographic grade formic acid, methanol, ethanol and acetonitrile were purchased from Sinopharm Chemical Reagent Co., Ltd. (Shanghai, China) and used for extractive and analytical procedures.

### 3.2. HPLC-Q-TOF-MS Conditions

Mass spectrum identification was performed on an Agilent 1290 HPLC system (Agilent Technologies, Palo Alto, CA, USA) combined with an accurate Agilent 6530 Q-TOF-MS mass spectrometer. Chromatographic separations used an XAqua C18 (2.1 × 150 mm, 5 μm, Agilent Technologies, Acchrom Technologies Co., Ltd, Beijing, China), the mobile phase A was deionized water (0.1% formic acid), and the mobile phase B was acetonitrile. The injection volume was 10 μL and the flow rate was 0.7 mL/min. The detection wavelength was set to 284 nm and the column temperature was maintained at 30 °C. The gradient elution procedure was optimized as follow: 0~10 min, 0~3.5% B; 10~13 min, 3.5~5% B; 13~18 min, 5~15% B; 18~21 min, 15~17.5% B; 21~32 min, 17.5~22% B; 32~44 min, 22~31% B; 44~58 min, and 31~90% B. Then, the effluent of the HPLC mobile phase was split (10:1) and guided into the electrospray ionization (ESI) source. MS conditions were performed as follow: Capillary voltage, 3500 V; nebulizer pressure, 50 psi; nozzle voltage, 1000 V; flow rate of drying gas, 6 L/min; temperature of sheath gas, 350 °C; flow rate of sheath gas, 11 L/min; skimmer voltage, 65 V; OCT1 RF Vpp, 750 V; fragmentor voltage, 135 V. The mass information was recorded in the range of *m*/*z* 100–1000 Da. The MS^2^ data of the selected targets were analyzed by regulating multilevel collision energy (18–45 eV). Data handling of the chemical compounds of AF was analyzed by the Mass Hunter Qualitative Analysis B.04.00.

### 3.3. GC-MS Conditions

GC-MS analysis of volatile oils was performed on an Agilent 6850 Network GC System coupled to an Agilent 5975C VL Mass Selective Detector (MSD). An HP-5 MS capillary column (30.0 m × 250 μm × 0.25 μm) was used for the separation. Helium (purity 99.99%) was employed as the carrier gas, with a flow rate of 3.0 mL/min. The splitting ratio was set at 10:1. The injection volume was 1.0 μL and the interface temperature to 250 °C. The MS source temperature was set to 230 °C and the MS quadrupole temperature to 150 °C. The mass spectra plot was acquired using the full scan monitoring mode with a mass scan range of *m*/*z* 45–450. The column temperature was initially set at 50 °C (3 min held) and programmed to rise at 5 °C/min to 220 °C (3 min held), 5 °C/min to 250 °C (3 min held), and 5 °C/min to 310 °C (3 min held).

### 3.4. HPLC Quantitative Determination Conditions

An Agilent 1260 HPLC system with a ZORBAX SB-C18 column (4.6 mm × 250 mm, 5 μm, Agilent Technologies, Palo Alto, CA, USA) was employed for the determination of chemical markers in AF. The mobile phases, injection volume, flow rate, detection wavelength and column temperature were consistent with Section 3.2, but with a little difference in the elution procedure as follows: 0~5 min, 10~15% B; 5~10 min, 15~18% B; 10~15 min, 18~20% B; 15~20 min, 20~22% B; 20~25 min, 22~22% B; 25~30 min, 22~25% B; 30~32 min, 25~28% B; 32~35 min, 28~50% B; 35~38 min, 50~50% B; 38~40 min, 50~65% B; 40~43 min, 65~90% B; 43~48 min, 90~90% B; 48~50 min, 90~10% B; 50~55 min, 10% B.

### 3.5. Preparation of Samples and Standards

#### 3.5.1. Preparation of AF Extract (Ethanol Solution)

The conditions were optimized in our previous study via response surface methodology. In short, 50 mg of each AF sample was added to 20 mL of 58% ethanol, and extracted for 17 min in a 70 °C water bath by use of an ultrasonic instrument with a constant power (200 W, 40 Hz). After the process, the solution weight was re-adjusted and complement, then the extract solution was filtered through a 0.22 μm membrane for further direct Q-TOF-MS analysis and HPLC determination. This extraction procedure was fast, low energy, low solvent consumption, and low toxicity, and could be considered to be an economical preparation method. 

#### 3.5.2. Preparation of AF Extract (Volatile Oils)

A 500 g AF sample was added into the supercritical fluid extraction device. The conditions of the device included temperature (50 °C), pressure (20 Mpa), time (2 h) and flow of CO_2_ (2 L/h), and were improved. Then, the volatile oils were sealed immediately for direct GC-MS analysis. This extraction method was a friendly procedure with low volatility and no toxicity.

#### 3.5.3. Preparation of Mixed Standard Solution

The standards of eriocitrin (0.39 mg), neoeriocitrin (0.38 mg), narirutin (0.41 mg), naringin (0.44 mg), hesperidin (0.37 mg), neohesperidin (0.42 mg), poncirin (0.44 mg), naringenin (0.44 mg), hesperetin (0.45 mg), nobiletin (0.39 mg), tangeretin (0.38 mg), and auraptene (0.36 mg) were dissolved in 0.5 mL of methanol to obtain the mixed standard solution. A series of concentration gradients were prepared by serial dilution and were filtered through a 0.22 μm membrane and directly determined.

### 3.6. Validation of Quantitative Analysis HPLC

To determine the limits of detection and quantification, methanol stock solution containing the 12 reference compounds was diluted into a series of appropriate concentrations with the same solvent, and aliquots of the diluted solutions were injected into HPLC for analysis. The limits of detection (LOD) and quantification (LOQ) under the present chromatographic conditions were determined at S/N (the ratios of signal to noise) of three and 10, respectively. Calibration curves were fitted by HPLC response for at least seven appropriate concentrations in triplicate of each markers. The precision was investigated by analyzing a proper calibration sample with the chromatographic system and repeated six times. For repeatability, the mixed solution was examined for six replicates within one day. To investigate the stability of the sample, the sample solution was analyzed at different time points (0, 1, 2, 4, 6, 12, 18, and 24 h). To evaluate the accuracy of the analytical method, the recovery study was measured by analyzing spiked samples. A certain amount of reference substances (low, medium, and high concentrations) were added into a certain amount of AF samples, respectively, and then were extracted and analyzed under the proposed method.

## 4. Conclusions

A sum of 104 compounds were identified or tentatively assigned in this study from ethanol extraction and volatile oils of AF by HPLC-Q-TOF-MS and GC-MS methods. The detailed chemical composition of AF was systematically illustrated and classified by comparing retention times, MS spectra of exact mass, fragmentation behaviors and data previously reported on this issue. Among them, 41 compounds were reported in AF for the first time. This provides the basic chemical data for further pharmacological and clinical research. Then, the direct quantitative determination of 12 biochemical markers for AF was efficiently established by the HPLC method that could be suitable for the quality control of AF. The main advantage of the proposed method is the low required amount of the sample, low organic solvents, and low energy consumption, from sample preparation to analytical procedures. Thus, it could be considered an efficient method for analysis and determination of AF samples [22,48]. We anticipate that this research will become an important basis for better comprehension of the pharmacological profiles and for practical application of the QA and QC of AF, which should be developed into a high quality traditional Chinese medicine and health functional foods in the future.

## Figures and Tables

**Table 1:** Identification of constituents from *Aurantii fructus* by the high-performance liquid chromatography quadrupole time-of-flight mass spectrometry method in positive and negative ion modes.

No.	T_R_ (min)	(ESI+)	(ESI−)	Fragmentations (ESI+/ESI−)	MW (Mea.)	MW (MFG)	Formula	Compound	Error ^b^ (ppm)
**Amino acids and peptides**									
1	1.221	147.1130	—	—/—	146.1057	146.1055	C_6_H_14_N_2_O_2_	Lysine ^c^	−1.25
2	1.329	147.0748	—	—/—	146.0495	146.0491	C_5_H_10_N_2_O_3_	Glutamine ^c^	−2.4
3	1.341	156.0761	—	—/—	155.0688	155.0695	C_6_H_9_N_3_O_2_	Histidine ^c^	4.42
4	1.372	175.1184	—	—/—	174.1111	174.1117	C_6_H_14_N_4_O_2_	Arginine ^c^	3.16
5	1.646	116.0709	—	—/—	115.0636	115.0633	C_5_H_9_NO_2_	Proline ^c^	−2.53
6	2.221	118.0863	—	—/—	117.0791	117.0790	C_5_H_11_NO_2_	Valine ^c^	−0.62
7	4.445	132.1013	—	—/—	131.0941	131.0946	C_6_H_13_NO_2_	Isoleucine ^c^	4.37
8	4.678	132.1016	—	—/—	131.0943	131.0946	C_6_H_13_NO_2_	Leucine ^c^	2.43
9	5.813	182.0807	—	—/—	181.0734	181.0739	C_9_H_11_NO_3_	Tyrosine ^c^	2.78
10	9.646	166.0862	—	—/—	165.0789	165.0790	C_9_H_11_NO_2_	Phenylalanine ^c^	0.54
11	17.321	205.0968	—	—/—	204.0895	204.0899	C_11_H_12_N_2_O_2_	Tryptophan ^c^	1.65
12	42.815	728.3966	—	—/—	727.3893	727.3905	C_36_H_53_N_7_O_9_	Citrusin III ^c^	1.59
13	47.024	704.3978	—	—/—	703.3905	703.3905	C_34_H_53_N_7_O_9_	Citrusin I ^c^	0.07
**Alkaloids**									
14	2.167	138.0549	—	—/—	137.0476	137.0477	C_7_H_7_NO_2_	Aminobenzoic acid ^c^	0.39
15	2.490	130.0865	—	—/—	129.0792	129.0790	C_6_H_11_NO_2_	Pipecolic acid ^c^	−1.82
16	2.865	144.1022	—	—/—	143.0950	143.0946	C_7_H_13_NO_2_	Piperidineacetic Acid ^c^	−2.33
17	6.876	168.1016	—	150.0853/—	167.0943	167.0946	C_9_H_13_NO_2_	Synephrine ^a^	1.98
18	8.344	152.1071	—	—/—	151.0998	151.0997	C_9_H_13_NO	*N*-methyltyramine	−0.88
19	9.262	268.1027	—	—/—	267.0955	267.0968	C_10_H_13_N_5_O_4_	Adenosine	4.65
20	16.402	196.0970	—	—/—	195.0897	195.0895	C_10_H_13_NO_3_	*N*-Acetylnorsynephrine	−0.98
**Flavanones**									
21	22.119	—	741.2261	—/—	742.2334	742.2320	C_33_H_42_O_19_	Naringenin -7-*O*-triglycoside	−1.79
22	24.235	597.1808	595.1663	451.1351, 289.0702/—	596.1736	596.1741	C_27_H_32_O_15_	Eriodictyol-7-*O*-rutinoside (Eriocitrin) ^a^	0.88
23	24.721	—	449.1101	—/—	450.1174	450.1162	C_21_H_22_O_11_	Eriodictyol-7-*O*-glucoside ^c^	−2.54
24	25.170	597.181	595.1656	451.1353, 289.0701/—	596.1742	596.1741	C_27_H_32_O_15_	Eriodictyol-7-*O*-neohesperidoside (Neoeriocitrin) ^a^	−0.07
25	27.442	581.1858	579.1705	435.1278, 419.1330, 273.0753/—	580.1785	580.1792	C_27_H_32_O_14_	Naringenin-7-O-rutinoside (Narirutin) ^a^	1.21
26	28.694	581.1864	579.1687	435.1285, 419.1327, 273.0755/—	580.1791	580.1792	C_27_H_32_O_14_	Naringenin-7-*O*-neohesperidoside (Naringin) ^a^	0.13
27	29.021	435.1286	—	273.0751/—	434.1213	434.1213	C_21_H_20_O_10_	Naringenin-7-*O*-glucoside	0
28	29.633	755.2392	753.2233	—/—			C_34_H_42_O_19_	Brutieridin ^c^	
29	29.746	611.1968	609.1803	449.1431, 347.0762, 303.0858/—	610.1895	610.1898	C_28_H_34_O_15_	Hesperetin-7-O-rutinoside (Hesperidin) ^a^	0.48
30	31.116	611.1965	609.1811	449.1432, 303.0856/—	610.1892	610.1898	C_28_H_34_O_15_	Hesperetin-7-*O*-neohesperidoside (Neohesperidin) ^a^	0.98
31	31.726	465.1381	—	303.0861/—	464.1308	464.1319	C_22_H_24_O_11_	Hesperetin-7-*O*-glucoside	2.24
32	36.794	289.0707	287.0546	—/—	288.0634	288.0634	C_15_H_12_O_6_	Eriodictyol	−0.08
33	39.559	595.2017	593.1875	433.1481, 287.0911/—	594.1944	594.1949	C_28_H_34_O_14_	Isosakuranetin-7-*O*-neohesperidoside (Poncirin) ^a^	0.79
34	41.245	449.1432	—	287.0898/—	448.1360	448.1369	C_22_H_24_O_10_	Isosakuranetin-7-*O*-glucoside (Isosakuranin) ^c^	2.19
35	43.417	273.0752	271.0610	—/—	272.0680	272.0685	C_15_H_12_O_5_	Naringenin ^a^	1.84
36	44.319	725.2285	723.2127	—/—	724.2212	724.2215	C_33_H_40_O_18_	Melitidin ^c^	0.35
37	45.613	303.0860	301.0730	—/—	302.0788	302.0790	C_16_H_14_O_6_	Hesperetin ^a^	0.9
38	50.531	287.0911	—	—/—	286.0838	286.0841	C_16_H_14_O_5_	Isosakuranetin	1.16
**Flavones**									
39	20.435	611.1598	—	465.0882, 303.0529/—	610.1526	610.1534	C_27_H_30_O_16_	Quercetin-3-*O*-rutinoside (Rutin) ^a^	1.29
40	21.337	595.1663	593.1500	—/—	594.1590	594.1585	C_27_H_30_O_15_	Isovitexin-7-*O*-glucoside (Saponarin)	−0.96
41	21.947	625.1762	—	—/—	624.1690	624.1690	C_28_H_32_O_16_	Diosmetin-6,8-*di*-*C*-glucoside	0.12
42	22.406	625.1750	—	—/—	624.1627	624.1690	C_28_H_32_O_16_	Diosmetin-6,8-*di*-*C*-glucoside isomer ^c^	2.03
43	23.919	741.2213	—	595.1647, 271.0617/—	740.2141	740.2164	C_33_H_40_O_19_	Apigenin-7-*O*-rutinoside-4′-*O*-glucoside ^c^ (Isorhoifolin-4′-*O*-glucoside)	3.2
44	26.856	463.1240	461.1089	301.0707/—	462.1167	462.1162	C_22_H_22_O_11_	Diosmetin-7-*O*-glucoside ^c^	−1.0
45	27.066	595.1657	—	287.0559/—	594.1584	594.1585	C_27_H_30_O_15_	Luteolin-7-*O*-rutinoside (Veronicastroside) ^c^	0.06
46	30.431	579.1704	577.1549	271.0596/—	578.1631	578.1636	C_27_H_30_O_14_	Apigenin-7-*O*-rutinoside (Isorhoifolin)	0.7
47	31.776	609.1802	—	301.0701/—	608.1730	608.1741	C_28_H_32_O_15_	Diosmetin-7-*O*-rutinoside (Diosmin)	1.89
48	31.594	609.1807	—	301.0716/—	608.1734	608.1741	C_28_H_32_O_15_	Diosmetin-7-*O*-neohesperidoside (Neodiosmin)	1.18
49	34.740	667.1866	—	301.0718/—	666.1793	666.1796	C_30_H_34_O_17_	Diosmetin-7-O-(6″-*O*-acetyl) neohesperidoside ^c^	0.41
50	36.519	579.1706	—	271.0600/—	578.1633	578.1636	C_27_H_30_O_14_	Apigenin-7-*O*-neohesperidoside (Rhoifolin)	0.36
51	41.772	697.1964	—	—/—	696.1893	696.1902	C_31_H_36_O_18_	Isovitexin-7-*O*-xylocoside-2″-*O*-arabinoside ^c^	1.29
52	46.08	271.0602	—	—/—	270.0529	270.0528	C_15_H_10_O_5_	Apigenin	−0.37
**Polymethoxyflavonoids (PMFs)**									
53	47.191	329.1022	—	314.0785, 299.0589, 271.0517/—	328.0949	328.0947	C_18_H_16_O_6_	3-Hydroxy-5,7,8-trimethoxyflavone ^c^	−0.68
54	48.444	359.1126	—	344.0871, 326.0769, 298.0732/—	358.1053	358.1053	C_19_H_18_O_7_	5-Hydroxy-6,7,3′,4′ -tetramethoxyflavone	−0.08
55	49.145	389.1223	—	374.0963, 359.0822, 356.0817, 328.0793/—	388.1150	388.1158	C_20_H_20_O_8_	5-Hydroxy-6,7,3′,4′,5-pentamethoxyflavone ^c^	2.15
56	50.218	373.1271	—	358.1041, 343.0816, 315.0828/—	372.1299	372.1209	C_20_H_20_O_7_	5,7,8,3′,4′-Pentamethoxyflavone (Isosinensetin)	2.8
57	50.857	373.1279	—	358.1050, 343.0926, 312.0896/—	372.1206	372.1209	C_20_H_20_O_7_	5,6,7,3′,4′-Pentamethoxyflavone (Sinensetin)	0.88
58	51.475	389.1220	—	359.0811, 360.0783, 341.0698/—	388.1147	388.1158	C_20_H_20_O_8_	5-Hydroxy-6,7,8,3′,4′-pentamethoxyflavone (Demethylnobiletin)	2.76
59	51.717	403.1386	—	373.0926, 355.0857, 327.0835/—	402.1313	402.1315	C_21_H_22_O_8_	5,6,7,8,3′,4′ -Hexamethoxyflavone (Nobiletin) ^a^	0.45
60	51.851	433.1495	—	403.1023, 388.0882, 385.0899/—	432.1422	432.1420	C_22_H_24_O_9_	3,5,6,7,8,3′,4′-Heptamethoxyflavone	−0.46
61	52.001	343.1170	—	328.0943, 313.0831, 285.0757/—	342.1097	342.1103	C_19_H_18_O_6_	5,6,7,4′-Tetramethoxyflavone	1.74
62	52.135	419.1337	—	389.0973, 361.0911/—	418.1264	418.1264	C_21_H_22_O_9_	5-Hydroxy-3,6,7,8,3′,4′-hexamethoxyflavone ^c^	0.05
63	52.945	359.1129	—	329.0883, 298.0722/—	358.1056	358.1053	C_19_H_18_O_7_	5-Hydroxy-3,6,7,8-tetramethoxyflavone ^c^	−1.04
64	52.394	343.1171	—	313.0735, 285.0788, 282.0767/—	342.1198	342.1103	C_19_H_18_O_6_	5,7,8,4′-Tetramethoxyflavone	1.57
65	53.062	373.1276	—	358.1000, 343.0722, 325.0655, 297.0697/—	372.1203	372.1209	C_20_H_20_O_7_	5,6,7,8,4′-Pentamethoxyflavone (Tangeretin) ^a^	1.59
66	53.621	419.1334	—	390.0919, 389.0914, 371.0816/—	418.1261	418.1264	C_21_H_22_O_9_	3-Hydroxy-5,6,7,8,3′,4′-hexamethoxyflavone (Natsudaidai)	0.66
67	54.298	359.1117	—	330.0734, 329.0712, 311.0653/—	358.1044	358.1053	C_19_H_18_O_7_	5-Hydroxy-6,7,8,4′-tetramethoxyflavoned	2.29
**Triterpenoids**									
68	24.029	471.2006	—	—/—	470.1934	470.1941	C_26_H_30_O_8_	Limonin ^a^	1.53
69	24.621	—	649.2482	—/—	650.2554	650.2575	C_32_H_42_O_14_	Limonin-17-*β*-d-glucoside	3.15
70	32.610	—	693.2753	—/—	694.2825	694.2837	C_34_H_46_O_15_	Nominin-17-*β*-d-glucoside ^c^	1.65
71	32.878	—	651.1553	—/—	652.1626	652.1639	C_29_H_32_O_17_	Obacunoic acid-17-*β*-d-glucoside ^c^	2.08
72	33.619	—	711.2858	—/—	712.2930	712.2942	C_34_H_48_O_16_	Nomilinic acid-17-*β*-d-glucoside	1.68
73	49.631	471.2013	—	—/—	470.1940	470.1941	C_26_H_30_O_8_	Limonin isomer ^c^	
**Coumarins**									
74	33.899	301.1065	—	—/—	300.0992	300.0998	C_17_H_16_O_5_	Phellopterin	1.78
75	34.139	261.1119	—	—/—	260.1047	260.1049	C_15_H_16_O_4_	Meranzin ^c^	0.79
76	37.696	203.0342	—	—/—	202.0270	202.0266	C_11_H_6_O_4_	Xanthotoxol ^a^	4.29
77	39.529	287.0909	—	—/—	286.0836	286.0841	C_16_H_14_O_5_	Oxypeucedanin	1.85
78	45.872	193.0491	—	—/—	192.0419	192.0423	C_10_H_8_O_4_	Scopoletin ^c^	2.05
79	47.999	355.1522	—	—/—	354.1449	354.1467	C_21_H_22_O_5_	Epoxybergamottin	4.98
80	49.028	261.1118	—	—/—	260.1045	260.1049	C_15_H_16_O_4_	Isomeranzin	1.19
81	53.797	245.1171	—	—/—	244.1098	244.1099	C_15_H_16_O_3_	Osthol	0.42
82	55.112	299.1654	—	—/—	298.1581	298.1569	C_19_H_22_O_3_	Auraptene ^a^	−4.21
**Other compounds**									
83	3.206	—	191.0188	—/—	192.0261	192.0270	C_6_H_8_O_7_	Citric acid	4.57
84	11.989	127.0388	—	109.0289/—	126.0316	126.0317	C_6_H_6_O_3_	5-Hydroxymethyl furfual ^c^	1.11
85	24.460	481.1680	—	—/—	480.1607	480.1632	C_23_H_28_O_11_	Paeoniflorin ^c^	5.11
86	28.085	625.2100	—	—/—	624.2027	624.2054	C_29_H_36_O_15_	Magnoloside A	4.41

^a^ These compounds were accurately identified with reference standards; ^b^ Errors (ppm) were obtained by formula prediction software in the mass spectrometer; ^c^ These compounds were identified in *Aurantii fructus* for the first time. T_R_ = Retention time; MW (Mea.) = Molecular weight (measured); MW (MFG) = Molecular weight (molecular formula generated).

**Table 2:** Identification of 18 aromatic constituents from *Aurantii fructus* by the GC-MS method.

No.	T_R_ (min)	Compound	Formula	Relative Amount (%)
87	3.522	*p*-Xylene	C_8_H_10_	0.6
88	5.031	(−)-*α*-Pinene (−)-*α*-Pinene	C_10_H_16_	2.0
89	6.970	α-Phellandrene	C_10_H_16_	1.5
90	7.128	3-Carene 3-Carene	C_10_H_16_	0.4
91	7.558	Benzene	C_6_H_6_	0.8
92	7.575	d-Limonene	C_10_H_16_	43.1
93	8.318	Ocimene	C_10_H_16_	0.6
94	8.567	4-Carene 4-Carene	C_10_H_16_	5.0
95	9.431	Cyclohexene	C_6_H_10_	0.6
96	9.831	Linalool	C_10_H_18_O	26.4
97	12.382	Terpineol	C_10_H_18_O	2.9
98	15.377	Thymol ^c^	C_10_H_14_O	2.0
99	17.425	Limonene oxide	C_10_H_14_O	0.1
100	17.581	Copaene ^c^ *α*-Copaene	C_15_H_24_	1.0
101	20.222	1.6-Cyclodecadiene ^c^	C_10_H_16_	7.9
102	21.423	Naphthalene	C_10_H_8_	1.4
103	22.159	(+)-Aromadendrene ^c^ (+)-Aromadendrene	C_15_H_24_	0.8
104	24.807	2-Naphthalenemethanol	C_11_H_10_O	3.0

^c^ These compounds were identified in Aurantii fructus for the first time.

**Table 3:** Linearity, LOD, LOQ, precision, repeatability, stability and recovery of the 12 chemical markers.

Chemical Markers	Regression Equation	Linearity (μg/mL)	LOD (μg/mL)	LOQ (μg/mL)	r	Precision RSD (%)	Repeatability RSD (%)	Stability RSD (%)	Recovery (mean ± SD)
Eriocitrin	*Y* = 17.787*X* + 0.7195	0.19–39.00	0.06	0.15	0.9999	1.12	0.93	3.17	101.35 ± 1.02
Neoeriocitrin	*Y* = 14.754*X* - 3.5435	0.38–190.00	0.09	0.33	0.9995	1.65	1.87	3.38	101.89 ± 0.33
Narirutin	*Y* = 25.375*X* + 13.516	0.41–205.00	0.11	0.40	0.9998	1.56	0.84	0.19	102.36 ± 0.21
Naringin	*Y* = 18.107*X* + 9.3102	0.44–880.00	0.12	0.38	0.9998	1.97	0.89	0.21	100.98 ± 0.87
Hesperidin	*Y* = 15.396*X* + 3.6443	0.74–740.00	0.21	0.67	0.9997	1.40	0.83	0.20	102.95 ± 1.21
Neohesperidin	*Y* = 26.069*X* + 10.197	0.42–840.00	0.10	0.39	0.9999	1.23	0.83	0.09	101.05 ± 1.92
Poncirin	*Y* = 17.126*X* + 5.1686	0.11–88.00	0.03	0.09	0.9995	0.87	0.72	0.26	100.77 ± 0.65
Naringenin	*Y* = 37.591*X* + 5.2889	0.06–22.00	0.02	0.06	0.9999	0.86	1.41	0.26	98.35 ± 0.55
Hesperetin	*Y* = 47.777*X* + 4.2667	0.06–22.50	0.02	0.06	0.9999	0.83	1.99	1.98	99.07 ± 0.73
Nobiletin	*Y* = 38.995*X* + 2.8379	0.20–39.00	0.05	0.18	0.9999	0.86	2.58	2.23	102.22 ± 1.24
Tangeretin	*Y* = 41.882*X* + 5.1345	0.19–38.00	0.05	0.17	0.9999	0.90	3.50	0.38	101.99 ± 0.90
Auraptene	*Y* = 11.111*X* + 1.7305	0.18–36.00	0.04	0.16	0.9999	0.81	1.13	1.29	98.36 ± 1.59

## Supplementary Materials

The following are available online at http://www.mdpi.com/1420-3049/23/9/2189/s1, Figure S1: Structures of 86 compounds form ethanol extract of *Aurantii fructus*. Figure S2: Structures of 18 compounds in volatile oils of *Aurantii fructus*. Table S1: 11 samples of *Aurantii fructus* obtained from different habitats. Table S2: Contents of chemical markers in *Aurantii fructus* from11 different samples (mg/g).
