# Supplementary material for: Chemical Profiles and Simultaneous Quantification of Aurantii fructus by Use of HPLC-Q-TOF-MS Combined with GC-MS and HPLC Methods
Source: Molecules. 2018 Aug 30;23(9):2189. doi: 10.3390/molecules23092189 (PMC6225099; doi:10.3390/molecules23092189)
Supplement: Supplementary file 1 [file molecules-23-02189-s001.pdf]

Supplementary Information

Chemical Profiles and Simultaneous Quantification of *Aurantii Fructus* by Use of HPLC-Q-TOF-MS Combined with GC-MS and HPLC Methods

Yingjie He [1,2,†], Zongkai Li [3,†], Wei Wang [2], Suren R. Sooranna [4], Yiting Shi [2], Yun Chen [2], Changqiao Wu [2], Jianguo Zeng [1,2], Qi Tang [1,2,*] and Hongqi Xie [1,2,*]

1 Hunan Key Laboratory of Traditional Chinese Veterinary Medicine, Hunan Agricultural University, Changsha 410128, China; E-Mails: yingjiehe272@163.com (Y.H.); zengjianguo@hunau.edu.cn (J.Z.)

2 National and Local Union Engineering Research Center for the Veterinary Herbal Medicine Resources and Initiative, Hunan Agricultural University, Changsha 410128, China; E-Mails: 18390946378@163.com (W.W.); erin643747964@163.com (Y.S.); m18250068565_1@163.com (Y.C.); m15207494911@163.com (C.W.)

3 School of Medicine, Guangxi University of Science and Technology, Liuzhou 565006, China; E-Mail: m13657294483_1@163.com (Z.L.)

4 Department of Surgery and Cancer, Chelsea and Westminster Hospital, Imperial College London, London SW10 9NH, UK; E-Mail: s.sooranna@imperial.ac.uk.

* Correspondence: tangqi@hunau.edu.cn (Q.T.); xiehongqi2006@163.com (H.X.); Fax: +86-0731-8461-5293

† These authors contributed equally to this work.

Amino acids

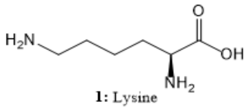

1: Lysine

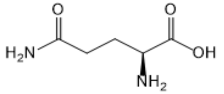

2: Glutamine

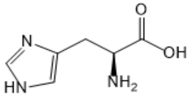

3: Histidine

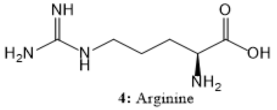

4: Arginine

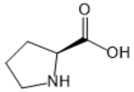

5: Proline

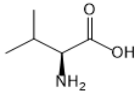

6: Valine

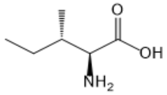

7: Isoleucine

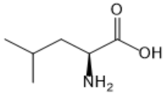

8: Leucine

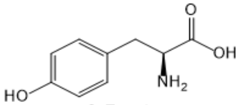

9: Tyrosine

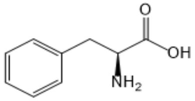

10: Phenylalanine

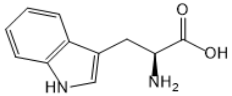

11: Tryptophan

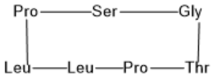

12: Citrusin III

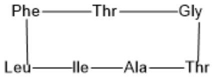

13: Citrusin I

Alkaloids

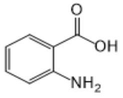

14: Aminobenzoic acid

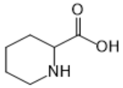

15: Pipecolic acid

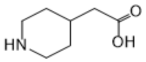

16: Piperidineacetic Acid

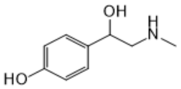

17: Synephrine

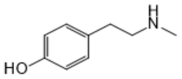

18: *N*-methyltyramine

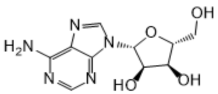

19: Adenosine

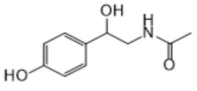

20: *N*-Acetylnorsynephrine

Flavanones

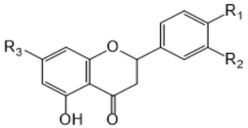

21: Naringenin-7-*O*-triglycoside; R_1=OH, R_2=H, R_3= -*O*-triglycoside
22: Eriodictyol-7-*O*-rutinoside; R_1=OH, R_2=OH, R_3= -*O*-rutinoside
23: Eriodictyol-7-*O*-glucoside; R_1=OH, R_2=OH, R_3= -*O*-glucoside
24: Eriodictyol-7-*O*-neohesperidoside; R_1=OH, R_2=OH, R_3=-*O*-neohesperidoside
25: Naringenin-7-*O*-rutinoside; R_1=OH, R_2=H, R_3= -*O*-rutinoside
26: Naringenin-7-*O*-neohesperidoside; R_1=OH, R_2=H, R_3= -*O*-neohesperidoside
27: Naringenin-7-*O*-glucoside; R_1=OH, R_2=H, R_3= -*O*-glucoside
28: Brutieridin; R_1=OCH_3, R_2=OH, R_3= -*O*-[2''-*alpha*-rhamnosyl-6''-(3''''-hydroxy-3''''-methylglutaryl)-*beta*-glucoside]
29: Hesperetin-7-*O*-rutinoside; R_1=OCH_3, R_2=OH, R_3= -*O*-rutinoside
30: Hesperetin-7-*O*-neohesperidoside; R_1=OCH_3, R_2=OH, R_3= -*O*-neohesperidoside
31: Hesperetin-7-*O*-glucoside; R_1=OCH_3, R_2=OH, R_3= -*O*-glucoside
32: Eriodictyol; R_1=OH, R_2=OH, R_3=OH
33: Isosakuranetin-7-*O*-neohesperidoside; R_1=OCH_3, R_2=H, R_3=-*O*-neohesperidoside
34: Isosakuranetin-7-*O*-glucoside; R_1=OCH_3, R_2=H, R_3= -*O*-glucoside
35: Naringenin; R_1=OH, R_2=H, R_3=OH
36: Melitidin; R_1=OH, R_2=H, R_3= -*O*-[2''-*alpha*-rhamnosyl-6''-(3''''-hydroxy-3''''-methylglutaryl)-*beta*-glucoside]
37: Hesperetin; R_1=OCH_3, R_2=OH, R_3=OH
38: Isosakuranetin; R_1=OCH_3, R_2=H, R_3=OH

Flavonoids

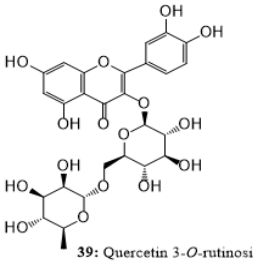

39: Quercetin 3-*O*-rutinoside

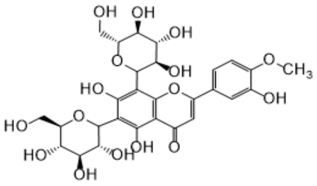

41: Diosmetin-6,8-di-*C*-glucoside
42: Diosmetin-6,8-di-*C*-glucoside (isomer)

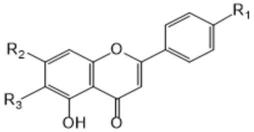

43: Apigenin-7-*O*-rutinoside-4'-*O*-glucoside; R_1= -*O*-glucoside, R_2= -*O*-rutinoside, R_3=H
46: Apigenin-7-*O*-rutinoside; R_1= OH, R_2= -*O*-rutinoside, R_3=H
50: Apigenin-7-*O*-neohesperidoside; R_1= OH, R_2= -*O*-neohesperidoside, R_3=H
52: Apigenin; R_1= OH, R_2= OH, R_3=H
40: Isovitexin-7-*O*-glucoside; R_1= OH, R_2= -*O*-glucoside, R_3=-*C*-glucoside
51: Isovitexin-7-*O*-xylocoside 2''-*O*-arabinoside; R_1= OH, R_2=-7-*O*-xylocoside 2''-*O*-arabinoside, R_3= -*C*-glucoside

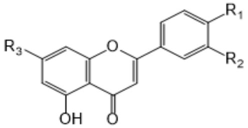

44: Diosmetin-7-*O*-glucoside; R_1=OCH_3, R_2=OH, R_3= -glucoside
45: Luteolin-7-*O*-rutinoside; R_1=OH, R_2=OH, R_3= -*O*-rutinoside
47: Diosmetin-7-*O*-rutinoside; R_1=OCH_3, R_2=OH, R_3= -*O*-rutinoside
48: Diosmetin-7-*O*-neohesperidoside; R_1=OCH_3, R_2=OH, R_3= -*O*-neohesperidoside
49: Diosmetin-7-*O*-(6''-*O*-acetyl) neohesperidoside; R_1=OCH_3, R_2=OH, R_3= -*O*-(6''-*O*-acetyl) neohesperidoside

Figure S1. Structures of 86 compounds form ethanol extract of *Aurantii fructus*.

Figure S1. Cont.

Polymethoxyflavonoids

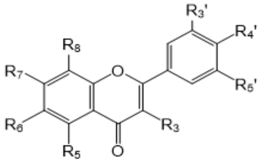

53: 3-Hydroxy-5,7,8-trimethoxyflavone; R_3=OH, R_5=OCH_3, R_6=H, R_7=OCH_3, R_8=OCH_3, R_3' =H, R_4' =H, R_5' =H
54: 5-Hydroxy-6,7,3',4'-tetramethoxyflavone; R_3=H, R_5=OH, R_6=OCH_3, R_7=OCH_3, R_8=H, R_3' =OCH_3, R_4' =OCH_3, R_5' =H
55: 5-Hydroxy-6,7,3',4',5'-pentamethoxyflavone; R_3=H, R_5=OH, R_6=OCH_3, R_7=OCH_3, R_8=H, R_3' =OCH_3, R_4' =OCH_3, R_5' =OCH_3
56: 5,7,8,3',4'-Pentamethoxyflavone; R_3=H, R_5=OCH_3, R_6=H, R_7=OCH_3, R_8=OCH_3, R_3' =OCH_3, R_4' =OCH_3, R_5' =H
57: 5,6,7,3',4'-Pentamethoxyflavone; R_3=H, R_5=OCH_3, R_6=OCH_3, R_7=OCH_3, R_8=H, R_3' =OCH_3, R_4' =OCH_3, R_5' =H
58: 5-Hydroxy-6,7,8,3',4'-pentamethoxyflavone; R_3=H, R_5=OH, R_6=OCH_3, R_7=OCH_3, R_8=OCH_3, R_3' =OCH_3, R_4' =OCH_3, R_5' =H
59: 5,6,7,8,3',4'-Hexamethoxyflavone; R_3=H, R_5=OCH_3, R_6=OCH_3, R_7=OCH_3, R_8=OCH_3, R_3' =OCH_3, R_4' =OCH_3, R_5' =H
60: 3,5,6,7,8,3',4'-Heptamethoxyflavone; R_3=OCH_3, R_5=OCH_3, R_6=OCH_3, R_7=OCH_3, R_8=OCH_3, R_3' =OCH_3, R_4' =OCH_3, R_5' =H
61: 5,6,7,4'-Tetramethoxyflavone; R_3=H, R_5=OCH_3, R_6=OCH_3, R_7=OCH_3, R_8=H, R_3' =H, R_4' =OCH_3, R_5' =H
62: 5-Hydroxy-3,6,7,8,3',4'-hexamethoxyflavone; R_3=OCH_3, R_5=OH, R_6=OCH_3, R_7=OCH_3, R_8=OCH_3, R_3' =OCH_3, R_4' =OCH_3, R_5' =H
63: 5-Hydroxy-3,6,7,8-tetramethoxyflavone; R_3=OCH_3, R_5=OH, R_6=OCH_3, R_7=OCH_3, R_8=OCH_3, R_3' =H, R_4' =H, R_5' =H
64: 5,7,8,4'-Tetramethoxyflavone; R_3=H, R_5=OCH_3, R_6=H, R_7=OCH_3, R_8=OCH_3, R_3' =H, R_4' =OCH_3, R_5' =H
65: 5, 6, 7, 8, 4'-Pentamethoxyflavone; R_3=H, R_5=OCH_3, R_6=OCH_3, R_7=OCH_3, R_8=OCH_3, R_3' =H, R_4' =OCH_3, R_5' =H
66: 3-Hydroxy-5,6,7,8,3',4'-hexamethoxyflavone; R_3=OH, R_5=OCH_3, R_6=OCH_3, R_7=OCH_3, R_8=OCH_3, R_3' =OCH_3, R_4' =OCH_3, R_5' =H
67: 5-Hydroxy-6,7,8,4'-tetramethoxyflavone; R_3=H, R_5=OH, R_6=OCH_3, R_7=OCH_3, R_8=OCH_3, R_3' =H, R_4' =OCH_3, R_5' =H

Triterpenoids

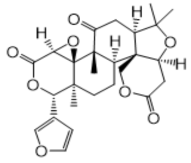

68: Limonin
73: Limonin isomer

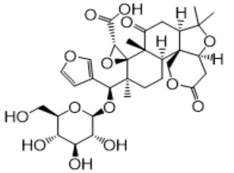

69: Limonin-17-*beta*-*D*-glucoside

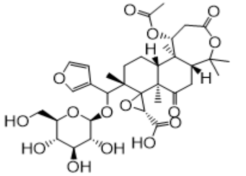

70: Nominin-17-*beta*-*D*-glucoside

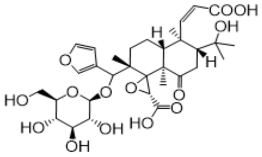

71: Obacunoic acid-17-*beta*-*D*-glucoside

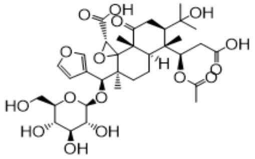

72: Nomilinic acid-17-*beta*-*D*-glucoside

Other compounds

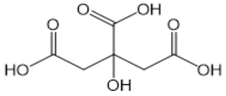

83: Citric acid

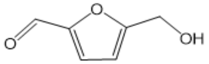

84: 5-hydroxymethyl furfural

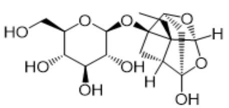

85: Paeoniflorin

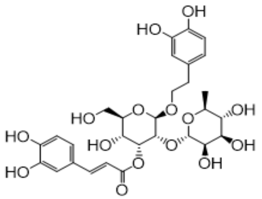

86: Magnoloside A

Coumarins

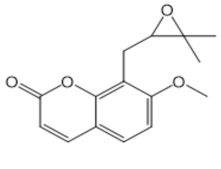

75: Meranzin
80: Meranzin isomer

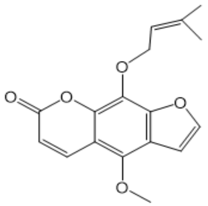

74: Phellopterin

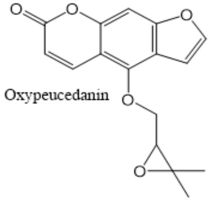

77: Oxypeucedanin

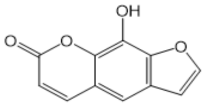

76: Xanthotoxol

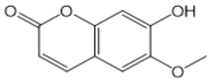

78: Scopoletin

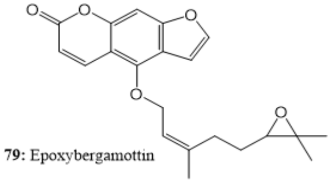

79: Epoxybergamottin

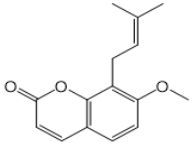

81: Osthol

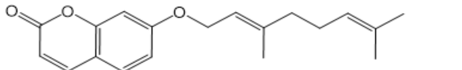

82: Auraptene

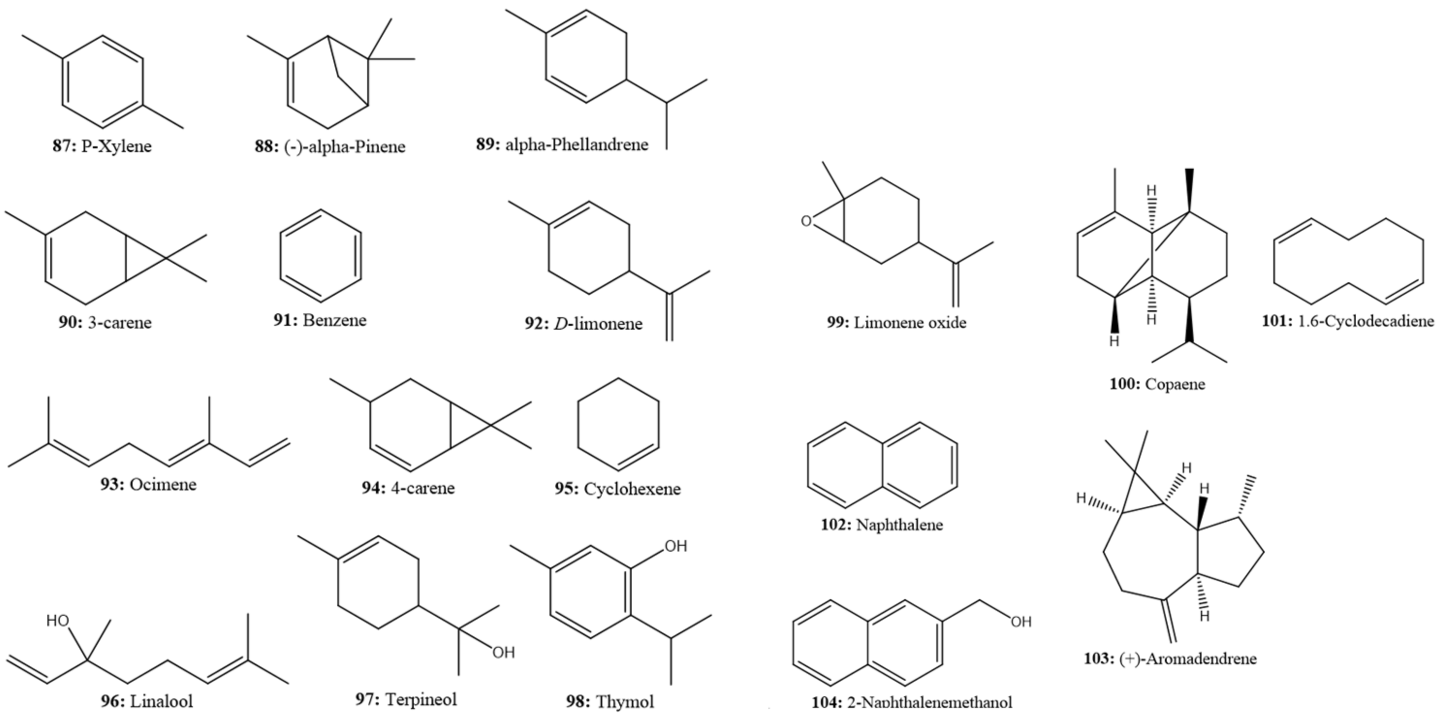

Figure S2. Structures of 18 compounds in volatile oils of *Aurantii fructus*.

Table S1. The 11 batches of *Aurantii fructus* obtained from different regions.

Sample	Region	Variety	Geographical location (approximate)	Collection date
S-1	Sanyantang, Yuanjiang, Hunan	*Citrus aurantium* L.	N28°45′, E112°21′, 30m	2016.06.30
S-2	Fuqiushan, Taojiang, Hunan	*Citrus aurantium* L.	N28°28′, E112°01′, 30m	2016.07.25
S-3	Hesanqu, Yiyang, Hunan	*Citrus aurantium* L.	N28°34′, E112°22′, 100m	2017.07.10
S-4	Yangjixiang, Anren, Hunan	*Citrus aurantium* L.	N26°38′, E113°13′, 80m	2016.07.06
S-5	Yangjixiang, Anren, Hunan	*Citrus aurantium* L.	N26°38′, E113°13′, 80m	2016.07.06
S-6	Hunan agricultural university	*Citrus aurantium* L.	N28°11′, E113°05′, 60m	2016.07.25
S-7	Fenglinzheng, Zhuzhou, Hunan	*Citrus aurantium* L.	N27°47′, E113°25′, 70m	2016.07.24
S-8	Shenzhengqiao, Xingan, Jiangxi	*Citrus aurantium* L.	N27°42′, E115°28′, 30m	2016.07.11
S-9	Shenzhengqiao, Xingan, Jiangxi	*Citrus aurantium* L.	N27°42′, E115°28′, 30m	2016.07.11
S-10	Zhangshu, Jiangxi	*Citrus aurantium* L.	N28°03′, E115°32′, 30m	2016.08.25
S-11	Changsha, Hunan	*Citrus aurantium* L. (variant)	N28°13′, E112°56′, 45m	2016.07.03

Table S2. Contents of chemical markers in *Aurantii fructus* from 11 different samples (mg/g)

**Batches**	**S1**	**S2**	**S3**	**S4**	**S5**	**S6**	**S7**	**S8**	**S9**	**S10**	**S11**
Eriocitrin	2.07	1.56	1.35	2.13	2.10	0.82	0.76	0.08	1.13	1.33	0.98
Neoeriocitrin	4.34	3.82	4.36	5.13	4.86	2.09	4.77	1.27	6.48	4.65	4.04
Narirutin	9.15	7.15	6.40	7.10	7.12	6.98	3.78	2.90	2.87	5.44	8.40
Naringin	106.81	101.97	97.59	96.91	98.44	82.35	96.13	100.68	89.98	92.35	80.40
Hesperidin	6.51	8.44	6.79	5.60	6.03	9.77	5.68	7.37	5.10	7.98	38.14
Neohesperidin	35.99	48.37	37.53	37.99	40.43	74.13	102.3	96.20	70.4	37.85	26.97
Poncirin	5.32	8.22	8.09	6.25	7.35	9.23	7.59	9.40	3.73	7.35	1.80
Naringenin	0.56	0.26	0.73	0.12	0.14	0.22	0.02	0.76	0.44	0.68	1.17
Hesperetin	0.13	0.12	0.20	0.18	0.19	0.15	0.05	0.38	0.24	0.28	0.57
Nobiletin	1.16	1.95	2.71	1.64	1.76	2.71	0.81	1.02	0.8	1.95	0.59
Tangeretin	1.39	2.29	2.90	1.81	1.85	1.90	0.78	1.30	0.81	2.28	0.43
Auraptene	1.22	1.71	2.13	1.36	1.67	0.42	0.43	0.15	0.35	2.00	0.36
Total (mg/g)	174.65	185.86	170.78	166.22	171.94	190.77	223.1	221.51	182.33	164.14	163.85
